# The Communion Cup and Infection

**Published:** 1918-12-14

**Authors:** 


*To the Editor of* The Hospital.

### The Communion Cup and Infection.

*To the Editor of* The Hospital.

Sir,—I desire to draw your attention to what I consider a dangerous custom in our religious services—*i.e.*, the Communion Service. I have often thought it such, more particularly since the influenza epidemic has broken out amongst us. I note that in your account (see The Hospital, November 23) of an outbreak in a village, "the church has been open for service from 5.30 a.m., the Sacrament being administered on several days in the week, and is attended by many men and women on their way to work. When the influenza came it spread rapidly and the position became more distressing and heartrending every day." I know it is rarely that individual cups are used, but it seems to me to be the most important step to take to obtain these, in a more general way. What is a more likely source of infection than the passing of the same cup from mouth to mouth? Whilst we would not think of using the same cup, even in our home circle, we are expected to put our lips to a cup which has been passed from divers persons who may be infectious. Apart from the present epidemic, what of the risks of tuberculosis? Patients are advised to use a separate cup, knife and fork and spoon and keep to these, having them washed separately from the others; yet they come to church to partake of the same one or perhaps two cups. We shall soon have our Christmas season upon us, when most people make a special effort to attend this service. Need we wonder if there is a further recurrence? When we are

**The Editor's Letter-Box**—(*continued*).

asked to take the cup, I do not think that it necessarily means a single cup; and can we reasonably expect the present epidemic to die down or disease to be stamped out whilst this custom prevails? If it is a question of cost, I suggest that each communicant should provide the cup or wine-glass, which I am sure they would willingly do, for their own safety. For my own part, I do not intend to take part in a Communion Service at the present time, unless individual cups are in use at the church, and should like to advise others to do likewise, but perhaps I might get in trouble with the clergy.—Yours truly,

HEALTH VISITOR AND A COMMUNICANT.

December 3, 1918.

[The idea of a separate cup is manifestly impracticable in many churches, but it is a custom now in some well-regulated churches for the officiating minister to wipe the cup after use by each communicant.—ED. THE HOSPITAL.]

[*We regret, owing to pressure on our space, some letters are unavoidably held over, including one in reference to "A Patient's Protest," and "The Increasing Terrors of Each Night."*]

